# Tracking Lymphatic Drainage Pathways Through Inner Ear Channels: A Systematic Review

**DOI:** 10.7759/cureus.66670

**Published:** 2024-08-12

**Authors:** Surraj Susai, Rohini Motwani, Mrudula Chandrupatla

**Affiliations:** 1 Department of Anatomy, All India Institute of Medical Sciences, Bibinagar, Hyderabad, IND

**Keywords:** network, vestibule, lymph, lacuna, cochlea

## Abstract

The search for potential lymphatic routes through the cochlea, or membranous portions of the inner ear labyrinth, remains a significant challenge. Researchers often focus on lower mammals rather than humans to uncover these pathways. This review aims to delineate the speculated lymphatic routes within the inner ear to date. It follows the Preferred Reporting Items for Systematic reviews and Meta-Analyses (PRISMA) guidelines, conducting a comprehensive search of PubMed, Scopus, Crossref, and Google databases using the terms “inner ear” and “lymph.” The problem, intervention, comparison, outcome (PICO) search strategy was employed, and analysis was performed using equation and scope metrics. Articles were screened and filtered using the CADIMA automation tool, resulting in 33 articles being reviewed, of which 23 were selected. Potential lymphatic drainage routes identified include the round window, oval window, scala tympani, spiral limbus, and lateral wall of the cochlea. The vestibular side of Reissner’s membrane was noted as a key nodal point for lymphocytes within the inner ear. This review maps the proposed lymphatic networks in the inner ear and highlights existing gaps. It systematically gathers, evaluates, and synthesizes available evidence on the lymphatic pathways of the inner ear, offering valuable insights into their presence, structure, function, and clinical significance.

## Introduction and background

The inner ear of humans is so complex in its structure that it has become almost impossible to imagine, locate, and trace a possible route to territorialize its lymphatic pathway to the external groups of lymph nodes, unlike its middle ear counterpart, which has had a well-defined sequential route of lymphatic drainage into its cervical exterior. The lymphatic system of the inner ear is a complex and somewhat contentious topic in otolaryngology. Historically, the inner ear was assumed to lack a conventional lymphatic system, which is responsible for draining the interstitial fluid and maintaining tissue fluid balance in most other organs of the body [1-4]. Over the years, many studies have been done in lower mammals such as guinea pigs, mice, and rats to find a possible lymphatic route through their inner ears, but each of those studies could not confidently derive a structured, well-set route from the starting point of a lymphatic portal up to its end, possibly due to the vivid entanglements and hurdles that existed during the course of their tracking paths [4-8]. Hence, this review aims to focus on the various possible lymphatic routes of the inner ear that had been observed in those animals and to subsequently extrapolate those findings to that of the human inner ear on an assumption basis, thereby helping the readers gain an insight into the myriad of possibilities that might exist in cracking the lymphatic code of the human inner ear, which has not been well understood yet.

## Review

Methodology

Following the Preferred Reporting Items for Systematic reviews and Meta-Analyses (PRISMA) guidelines, a thorough literature search was done from January 2024 to June 2024 using PubMed, Web of Science, and Google Scholar and using the following MeSH terms: inner ear AND lymph. The Boolean word “AND” was used instead of “OR” to link between the search terms as the authors wanted to include all relevant studies associated with lymphatic routes concerning the inner ear.

Inclusion Criteria

Only articles in English and published before June 2024 were included. Original studies associated with lymphatic routes concerning the inner ear were included.

Exclusion Criteria

Those studies that focused on the middle ear lymphatic route without giving impetus to the inner ear were excluded from the literature search. Case reports, letters to editors, and editorials were excluded. Also, articles whose full text was not available (no online access or the local library was unable to obtain a copy via library networks) and articles written in a language other than English without any translated English version were excluded.

Search Strategy and Method of Analysis

The problem, intervention, comparison, outcome (PICO) model search strategy was applied to this review using analytical methods of analysis by equation metrics and scope metrics.

Elimination of Bias

Three independent researchers reviewed and screened the articles for selection in this review, thereby eliminating selection bias. This review was also screened by the institutional review board to eliminate observer bias. Furthermore, the CADIMA automation tool was used to screen and filter the duplicate articles in order to eliminate technical bias.

**Figure 1:**
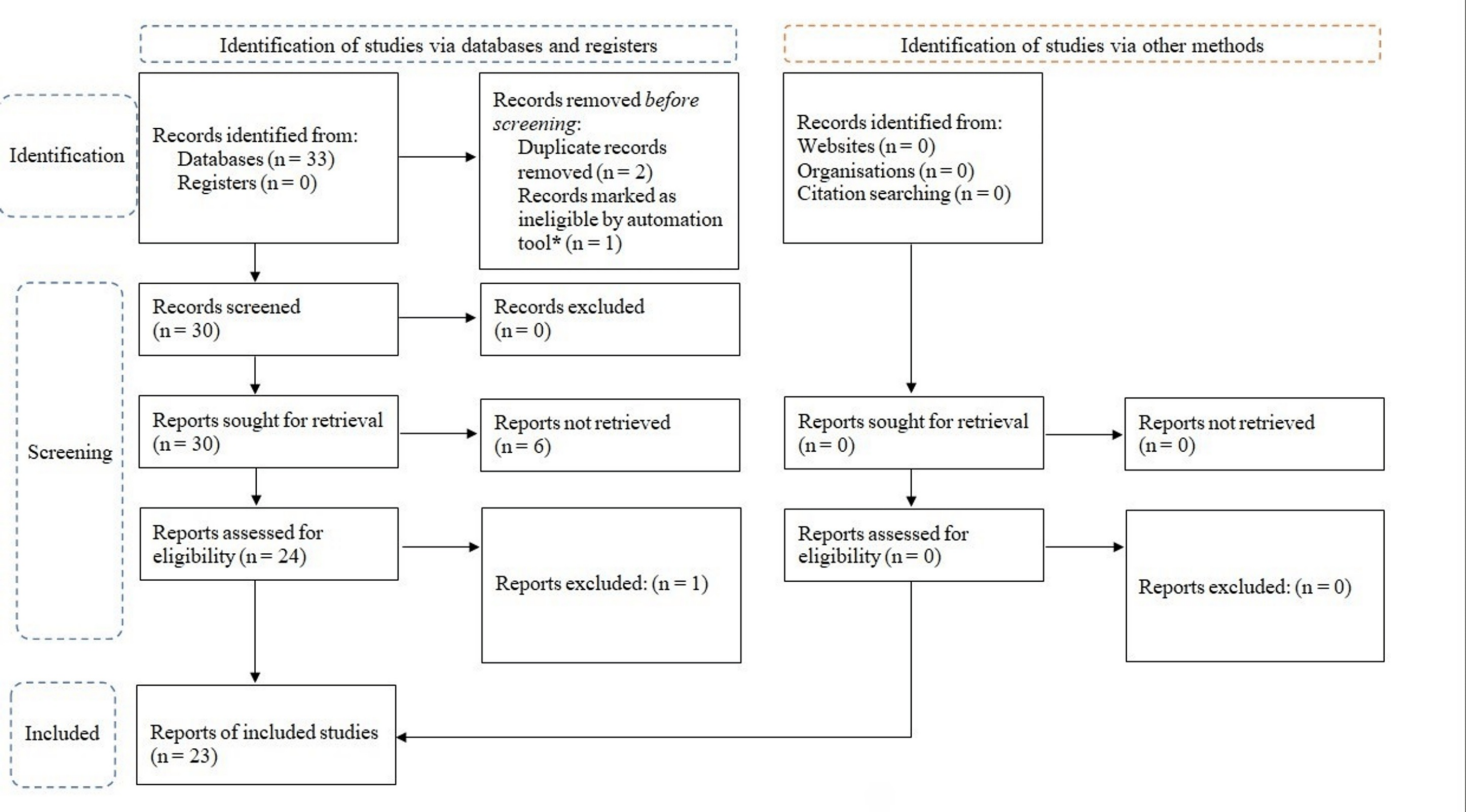
Methodology of the review following the PRISMA guidelines The CADIMA automation tool was utilized for screening and filtering. PRISMA, Preferred Reporting Items for Systematic reviews and Meta-Analyses

At the conclusion of the literature search, 33 relevant articles were identified from the databases regarding lymphatic routes through the inner ear. After removing duplicates, two reviewers independently screened the remaining 30 papers. Six articles were found to be unretrievable (Figure 1). The remaining 24 articles were assessed, and one was excluded for lacking specific data on inner ear lymphatic routes, as it only discussed hypothesized networks without substantial evidence. Thus, 23 articles were included in this systematic review (Figure 1). This review was approved by the Ethics Committee of the All India Institute of Medical Sciences, Bibinagar, Hyderabad, India (approval number AIIMS/BBN/IEC/EXEMPT/2023/359).

Review

Even though rats do have a cochlear structure similar to that of humans, guinea pigs were the preferred choice as subjects when it came to tracing the cochlear and vestibular lymphatics, possibly due to the fact that their fused ossicles offered a wider space within their capaciously large temporal bones so as to provide easy access to the seepage of tracer molecules through them, thereby aiding the researchers in their efforts to infuse radioactive tracers or antigens into the inner ears of those animals with effortless ease [1]. This became more evident owing to the fact that the cochleae, vestibules, and oval windows of guinea pigs were more permeable to the penetrative effects of antigens, thereby guaranteeing the researchers an effective antigenic platform for antibody interactions regarding the same in contrast to the inner ears of rats or mice [2-4]. Also, the effects of the blood-labyrinthine barrier in the fragile inner ears of the former led to the notion that they shared almost the same status as that of the central nervous system of the latter as far as immunity was concerned [3-5]. Currently, it is known that the inner ear has the ability to quickly develop an inflammatory response to viruses as well as an immunological response to foreign proteins that the animal might be sensitive to [4-6]. To defend against encroaching infections, immune-competent cells from the systemic circulation would not only invade the endolymphatic sac but also the perilymphatic compartments of the cochlea as well, despite the fact that the former would generally allow certain immune-competent cells to freely pass through it as opposed to the latter and would also accept antigens through diffusion from the perilymph, thereby undoubtedly helping to trigger an immune response [3-6]. The immune-competent cells that would infiltrate this arena would do so via systemic circulation, and this would naturally imply a link between the inner ear and the systemic environment with occult pathways between them that were traced by many workers using specialized molecules having selected affinities to specialized areas of the same [7,8].

Tracer Molecules

The substances that were frequently used by most of the researchers to trace the lymphatics of the inner ear included horseradish peroxidase (HRP), keyhole limpet hemocyanin (KLH), and radiolabeled lymphocytes [7,8]. The commonly employed HRP, being an exogenous protein for tracing the mammalian inner ear lymphatics, was unique in that it was easily transferred from the middle ear of guinea pigs to their inner ear counterparts by pinocytosis and was subsequently captured by the inner hair cells of their cochlear organ of Corti, through their basal ends, by endocytotic uptake, as a result of the selective affinities exhibited by these particles to their endoplasmic reticula and Golgi complexes [7]. The nutritional support and passage route for the reactionary end-products of these particles were provided by the perilymph [8]. KLH, on the other hand, is a mollusk-derived copper-enriched protein that was later synthesized artificially, and it eventually served as a replacement for HRP due to its potential immune-stimulant and immunomodulatory properties within the inner ear, as it was very effective in functioning as a cementing ladder for the purpose of routing the lymphocytes into the inner ear because of its heavy dependency on the T cells [9,10]. As of late, chromium-labeled lymphocytes have gained considerable significance as a tracing competitor for KLH [10,11]. These lymphocytes are sensitized in vitro using antigenic KLH and then subsequently tagged with a radioactive isotope of chromium 51. They were found to be more useful than KLH because of the promising benefits that were revealed by them from studies conducted on similarly tagged erythrocytes for the determination of red cell survival due to their sequential radioactive decays that were assessed while in the declining mode, and thus this property was eventually exploited by the researchers for the inner ear too, as a result of which they became a more reliable source for reaching the peripheral lymph nodes from the indexed area of the inner ear [10-14].

Traced Routes in Chronological Order

**Figure 2:**
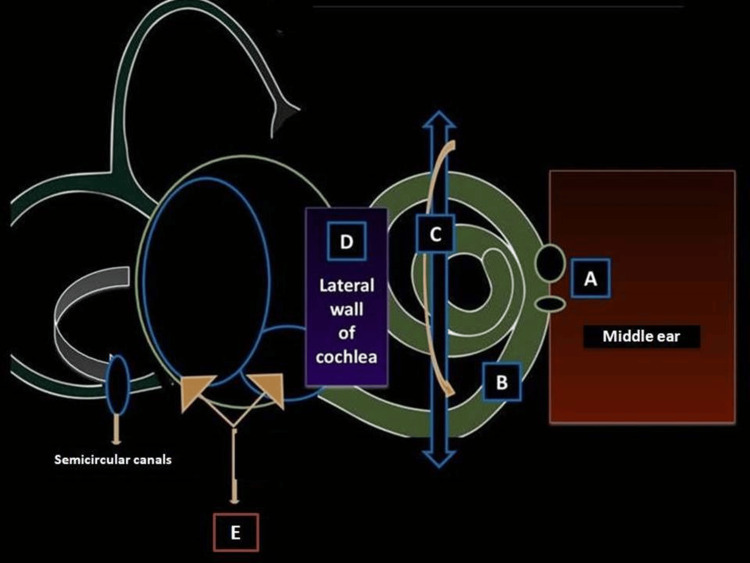
Possible routes of lymphatic drainage through the inner ear (A) Double-window route. (B) Scala tympani route. (C) Spiral limbus route. (D) Lateral cochlear route. (E) External linking route.

To facilitate readers’ understanding, the authors have outlined the traced lymphatic pathways of the inner ear in chronological order, based on research conducted in lower mammals (Figure 2). These pathways serve as guiding maps to identify crucial portals within the inner ear that might be involved in initiating the lymphatic network and connecting it to the external environment. The simplified diagram provided below (Figure 2) illustrates these pathways and their potential roles in the lymphatic system of the inner ear.

Double-window route: Saijo and Kimura observed in 1984 [15] that HRP particles that were infused into the middle ears of the guinea pigs reached their inner ears through the perilymph and subarachnoid spaces that were organized around the oval window rather than the round window. Subsequently, these particles had deposited heavily over that portion of Reissner’s membrane that faced the scala vestibule of the cochlear membranous labyrinth [15]. The high permeability of the oval window to these microparticles was postulated as the main reason behind this dense accumulation of HRP in the scala vestibuli, apart from the supposed perilymph route that was considered to have been the missing link for the passage of lymphatics into the inner ear from the oval window [15]. From Reissner’s membrane, the particles had managed to reach the endolymphatic sac via the vestibular aqueduct. Apart from the abovementioned pathway, Saijo and Kimura also deciphered later that the round window too had become permeable to the effects of HRP on repeated injections of the same, as a result of which the end-products of HRP had also reached the scala tympani in addition to the scala vestibuli through the round window, and eventually, these products had managed to reach the endolymphatic sac lumen [15].

Exclusive scala tympani route: The research conducted by Gloddek et al. in 1991 gave emphasis to the spiral modiolar vein as the main channelizer for the entry of lymphocytes, first into the basal turn of the scala tympani and then later into the other parts of the inner ear [1]. They also concluded that immune surveillance played a crucial role in the recruitment of lymphocytes into the inner ear from the peripheral circulation in response to the antigenic stimuli initiated by the sensitized radioactive chromium-labeled lymphocytes that were infused into the inbred guinea pigs. According to their study, the scala tympani was the site of the primary contact of the lymphocytes rather than the scala vestibuli, in partial contrast to the findings of Saijo and Kimura [15]. From the scala tympani, the labeled lymphocytes reached the endolymphatic sac lumen [1].

Spiral limbus to endolymphatic sac route: In 1995, Yeo et al. [16] observed from their study that the HRP particles accumulated densely throughout the cochlea two hours after injecting them into the scala tympani through the oval window. Within the cochlea, these particles were found to be clustered around the basilar membrane and spiral limbus rather than the stria vascularis. They then moved on to the lumen of the endolymphatic sac, where they were acted upon by the macrophages, which in turn paved the way for the further movement of HRP products from the spiral limbus toward the endolymphatic sac [16]. After 72 hours, it was observed that the products of HRP did not accumulate in the endolymph space of the cochlea but had managed to channel their way directly into the endolymphatic sac lumen through the perilymph and perisaccular connectivity network [16]. A similar replicated study was done in 1999 by Gong et al. [14], wherein they succeeded in detecting the radioactivities of right temporal bones from three distinct groups of radiochromium-sensitized guinea pigs. Their findings alluded to those of Gloddek et al., wherein they confirmed the peripheral route of entry of lymphocytes from the circulation after challenging the inner ears of interest with KLH in addition to their sensitization with radioisotopes [1].

Lateral cochlear portal route: According to the observations made by Ichimiya et al. in 2000 [17], when inflammatory stimuli were applied to the cochlea of guinea pigs either by artificially inducing membranous labyrinthitis with the help of KLH or by manipulating otitis media with the help of antigenic bacterial endotoxins, there was a facilitated process of priming of lymphocytes into the inner ear through the cochlear route as a result of the disruptions created by the recruitment of type 2 into the already existing bed of type 1 fibrocytes at the stria vascularis. The intermingling of these two varieties of fibrocytes within the spiral ligament, followed by their subsequent entanglement with the basal cells of the stria vascularis through declining gap junctions, created a ripple effect that led to the secretion of chemokines by the basal cells of the strial zone, which in turn served as a chemo-attractant for lymphocytes to be recruited into that area [17,18]. Their findings alluded to those of Gratton et al. [19], who had observed a similar phenomenon in gerbils wherein the type 1 fibrocytes of the stria vascularis were found to be the dominant force in pulling the lymphocytes, macrophages, and another type 2 fibrocytes toward them in the presence of weak gap junctions owing to the diminishing connexins in that area. The interactions of these two varieties of fibrocytes within the confines of the stria vascularis extending onto the spiral limbus were also observed and reported by Suko et al. [18], elucidating their role in the stability of the cochlear milieu. Hence, the above findings reveal that gap junctions between the basal cells of the strial complex at the lateral cochlear wall could serve as a portal for the channeling of lymphocytes into the inner ear in the presence of inflammatory triggers [17-19].

External linking route: In 2001, Yimtae et al. [13] were the first to demonstrate possible hypothetical evidence of a supposedly existing lymphatic connecting pathway from the inner ear of guinea pigs to the peripheral lymph nodes and lymphoid organs using immunohistochemical assays to detect the presence of KLH antigens using murine anti-KLH antibodies of a polyclonal nature in temporal bones, spleens, and lymph nodes perfused with KLH [13]. They concluded that perfusions into the middle ear produced identifiable labeled cells in the parotid, superficial ventral, mandibular, and deep cranial cervical lymph nodes, in contrast to the inner ear injections with KLH, wherein the KLH-labelled cells were present in only the parotid and superficial ventral cervical nodes. They also deduced that the spleen of those animals did contain cells positive for KLH following their injection into either the middle or inner ear. Thus, they inferred that the inner ear had its own connections with lymph nodes apart from its separate lymphatic connections with the middle ear [13]. Yimtae et al. had also proven that antibodies against KLH were inherently secreted by the cochlea in response to antigenic KLH, thereby confirming the possibility of the presence of residing B cells within the cochlea [13]. These findings were further strengthened by several other immunohistochemical and flow-cytometric studies done from the year 1999 onward, wherein many researchers confirmed the presence of lymphocytic markers in addition to the rampage of existing macrophages within the domains of the inner ear, which could possibly negate the possibility of the inner ear being an immune-privileged organ [20-23]. One such characteristic study in the year 2020 was done by Rai et al. [22], who showed in their study using flow cytometry that a heterogeneously heightened immune cell response of T cells and B cells was evoked on noise stimulation of the cochlea in rats. Their flow cytometric findings suggested the presence of immune cell markers, lymphocytic markers, and myeloid cell markers within the cochleae and vestibules of mice, thereby strongly suggesting the presence of a direct external lymphatic link from the inner ear to the peripheral external lymph nodes of mice; however, a continuity between the various paths leading to the exterior could not be established [22].

Results

Table 1 summarizes the methods employed by various researchers to explore these routes and the substances used to delineate them. The table also highlights the injection sites for these substances to access the inner ear. It is evident that most substances and their pathways through the inner ear are influenced by immune-mediated phenomena.

**Table 1:** Methods employed by researchers to identify potential lymphatic pathways in the inner ear CD45+T, cluster of differentiation 45 + thymic cells; HRP, horseradish peroxidase; KLH, keyhole limpet hemocyanin; Na-K ATPase, sodium-potassium-adenosine-tri-phosphatase

Researchers involved	Substance injected	Site of injection	Method of analysis	Interpreted route
Gloddek et al. (1991) [1]	Radiolabeled, sensitized lymphocytes	Cochlea of the right temporal bones, spleen, and cervical lymph nodes	Radioactive assay	Exclusive scala tympani route
Yimtae et al. (2001) [13]	KLH	Right scala tympani and right middle ear	Immunohistochemistry	Double-window route
Gong et al. (1999) [14]	Radiolabeled, sensitized lymphocytes	Right temporal bones	Radioactive assay	Spiral limbus to endolymphatic sac route
Saijo and Kimura (1984) [15]	HRP	Middle ear cavities, peri-lymphatic, and subarachnoid spaces	Estimation of HRP reaction products through chemical assay	Reissner’s membrane route of entry of lymphatics into the inner ear
Yeo et al. (1995) [16]	HRP	Scala tympani of the cochlear basal turn	Antigen presentation to macrophages	Spiral limbus to endolymphatic sac route
Ichimiya et al. (2000) [17]	KLH, albumin, connexin26, and Na-K ATPase	Scala tympani, spiral modiolar vein, and spiral limbus on both sides	Immunostaining	Lateral cochlear portal route
Rai et al. (2020) [22]	CD45+T cells	Bilateral cochlea	Flow cytometry and confocal imaging	External linkway

**Figure 3:**
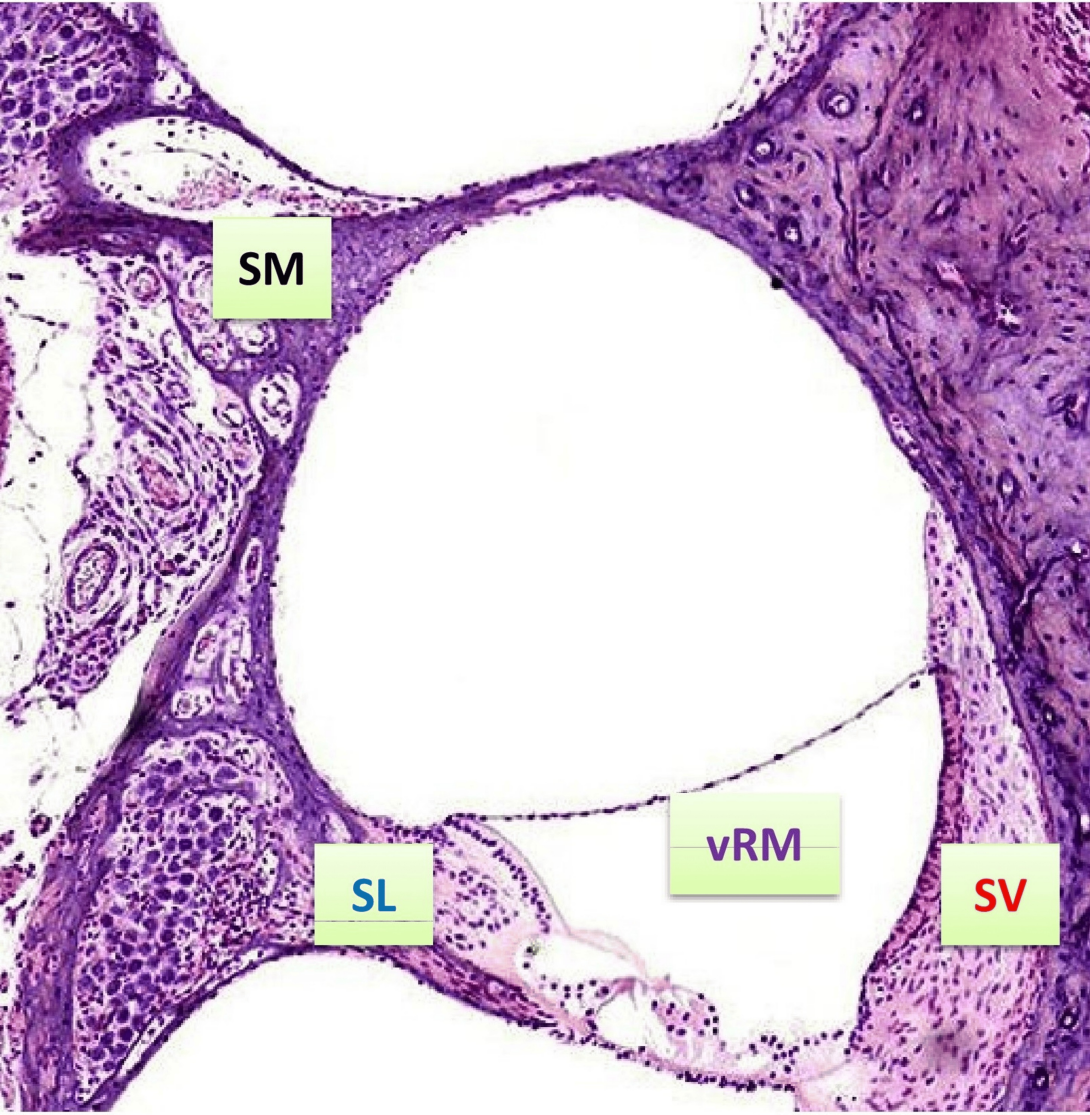
Microscopic image of a longitudinal cochlea section (40× magnification) highlighting potential nodal points for lymphocyte channeling (H&E stain) SL, spiral ligament; SM, spiral modiolus; SV, stria vascularis; vRM, vestibular side of Reissner’s membrane This microscopic slide of the cochlea was prepared, stained, and photographed by the first author.

Based on the observations and inferences from the above studies, the authors of this review have identified and deduced four possible nodal points within the cochlea of the inner ear (Figure 3) that could serve as sensors for the channeling of lymphocytes through the supposedly existing lymphatic pathways. These points include the spiral modiolus, the vestibular side of Reissner’s membrane, the stria vascularis, and the spiral limbus. These nodal points could not only serve as hotspots for future research concerned with the lymphatics of the inner ear but could also function as lymphatic inducers of the mammalian inner ears, owing to the fact that most of the antigenic interactions, immune-modulatory mechanisms, and lymphocytic drives are initiated at these areas [20-23]. Apart from the abovementioned four cochlear inducer zones, the authors would also like to suggest the possibility of two other extra cochlear zones that could serve either as promoters or as prompters for the lymphatic drive through the inner ear, and these would include the oval window and the peri-saccular connectivity network [2,7,21-23].

Future implications

This suggests that these inducer zones may either drive lymphocytes independently or in unison, depending on their activation state and the timing of their transit between portals [22,23]. The priming of lymphocytic antigenic receptors is crucial for directing this route, particularly if prior lymphocytic memory has familiarized them with the environment [23]. However, without the oval window and the perisaccular network, the likelihood of these nodal points triggering a strong response remains uncertain, which complicates tracing the lymphatic routes [7,16,23]. Understanding these lymphatic pathways in the inner ear could provide deeper insights into the pathophysiology of endolymphatic hydrops and help clarify whether Meniere’s disease is idiopathic [21-23]. It would also allow researchers to better define the role of brain lymphatics in endolymphatic hydrops and assess the causal relationships between them [23].

## Conclusions

Evidence from the literature indicates that priming the inner ears with sensitized antigens is crucial for tracking lymphocytes either from the cochleae or the inner membranous labyrinth to the exterior. To date, inherent lymphatic pathways independent of immunomodulation do not appear to exist, except in inner ears previously activated through polyclonal stimulation. Future multicentric trials are necessary to accurately identify the endpoints of the inner ear lymphatic routes.
